# Case report: A novel *de novo IGF2* missense variant in a Finnish patient with Silver-Russell syndrome

**DOI:** 10.3389/fped.2022.969881

**Published:** 2022-10-04

**Authors:** Petra Loid, Marita Lipsanen-Nyman, Sirpa Ala-Mello, Katariina Hannula-Jouppi, Juha Kere, Outi Mäkitie, Mari Muurinen

**Affiliations:** ^1^ Folkhälsan Research Center, Genetics Research Program, Helsinki, Finland; ^2^ Pediatric Research Center, Children's Hospital, University of Helsinki and Helsinki University Hospital, Helsinki, Finland; ^3^ Research Program for Clinical and Molecular Metabolism, University of Helsinki, Helsinki, Finland; ^4^ Department of Clinical Genetics, Helsinki University Hospital, Helsinki, Finland; ^5^ Department of Dermatology and Allergology, University of Helsinki and Helsinki University Hospital, Helsinki, Finland; ^6^ Stem Cells and Metabolism Research Program, University of Helsinki, Helsinki, Finland; ^7^ Department of Biosciences and Nutrition, Karolinska Institutet, Stockholm, Sweden; ^8^ Department of Molecular Medicine and Surgery, Karolinska Institutet, and Clinical Genetics, Karolinska University Hospital, Stockholm, Sweden

**Keywords:** IGF2, short stature, Silver-Russell, intrauterine growth restriction, exome sequencing

## Abstract

Silver-Russell syndrome (SRS, OMIM 180860) is a rare imprinting disorder characterized by intrauterine and postnatal growth restriction, feeding difficulties in early childhood, characteristic facial features, and body asymmetry. The molecular cause most commonly relates to hypomethylation of the imprinted 11p15.5 *IGF2*/*H19* domain but remains unknown in about 40% of the patients. Recently, heterozygous paternally inherited pathogenic variants in *IGF2*, the gene encoding insulin-like growth factor 2 (IGF2), have been identified in patients with SRS. We report a novel *de novo* missense variant in *IGF2* (c.122T > G, p.Leu41Arg) on the paternally derived allele in a 16-year-old boy with a clinical diagnosis of SRS. The missense variant was identified by targeted exome sequencing and predicted pathogenic by multiple *in silico* tools. It affects a highly conserved residue on a domain that is important for binding of other molecules. Our finding expands the spectrum of disease-causing variants in *IGF2*. Targeted exome sequencing is a useful diagnostic tool in patients with negative results of common diagnostic tests for SRS.

## Introduction

Silver-Russell syndrome (SRS, OMIM 180860) is a rare imprinting disorder characterized by intrauterine and postnatal growth restriction, feeding difficulties and/or low body mass index in early childhood, relative macrocephaly at birth, prominent forehead in infancy and body asymmetry (1). Clinical diagnosis of SRS can be made in patients who have at least four of the six abovementioned clinical features according to the Netchine-Harbison Clinical Scoring System (NH-CSS) (2). The estimated incidence of SRS is 1/75 000–1/100 000 (3).

*IGF2* is an imprinted gene expressed from the paternal allele, except in adult liver and central nervous system (4). *IGF2* encodes insulin-like growth factor 2 (IGF2), a hormone important for fetal growth and development. Hypomethylation of the imprinted 11p15.5 *IGF2*/*H19* domain leading to downregulation of *IGF2* is the main molecular cause of SRS (seen in 30%–60% of patients). Maternal uniparental disomy of chromosome 7 [UPD(7)mat] is another common cause of SRS (seen in 5%–10% of patients) (1, 5). However, among patients with SRS the molecular cause remains unknown in about 40% of the patients (5). Next generation sequencing has been proposed as a useful tool to increase the diagnostic yield in SRS (6–8). Recently, rare pathogenic/likely pathogenic single nucleotide variants (SNVs) and/or copy number variants (CNVs) involving *IGF2*, *CDKN1C*, *HMGA2*, *PLAG1* have been associated with SRS (6, 7, 9–18).

To date, only 16 pathogenic/likely pathogenic *IGF2* variants have been reported in patients with SRS- like phenotype (6, 8, 11–18). In this study, we report a novel *de novo* missense variant in *IGF2* identified by targeted exome sequencing in a Finnish patient with a clinical diagnosis of SRS. Our finding expands the spectrum of disease-causing variants in *IGF2*.

## Methods

This study was carried out at Children's Hospital, Helsinki University Hospital and Folkhälsan Research Center in Helsinki, Finland. This study is a part of a research project investigating genetic causes of growth restriction and Silver-Russell syndrome. The inclusion criteria for the study were: at least −2.5 standard deviation score (SDS) pre- and/or postnatal growth restriction, normal chromosomes, and growth restriction of unknown etiology. Participants with an established molecular diagnosis were excluded from the study. Written informed consents were obtained from all participants or their parents/guardians. Ethical approval for this study was obtained from the research ethics committee of the Hospital District of Helsinki and Uusimaa. We used a custom-made targeted exome sequencing panel including 566 genes associated with growth and skeletal disorders. The original raw data consist of a clinical exome sequencing assay of more than 4,000 genes, but only selected genes were analyzed. The gene panel includes all protein coding exons and 20 base pairs from exon-intron boundary. In addition, the panel includes known disease-associated non-coding, deep intronic variants and regulatory variants. Panel testing included sequence and copy number variation analyses of the 566 genes in the panel. The targeted exome sequencing was performed at Blueprints Genetics, Helsinki, Finland. Sequence reads were aligned to reference human genome (GRCh 37/hg19). Burrows-Wheeler Aligner software was used for read alignment and variant calling were performed using GATK. CNV analysis was performed by Blueprint Genetics Ltd. (a Quest clinical laboratory), using an in-house developed pipeline, which has been validated in the CLIA (Clinical Laboratory Improvement Amendments) and CAP (College of American Pathologists) accredited Blueprint Genetics diagnostic laboratory. Only regions covered by the panel were included in the CNV analysis. We performed Sanger sequencing to confirm the findings and to analyze parental samples. The HOPE tool (https://www3.cmbi.umcn.nl/hope) was used to predict the structural effect of amino acid change on protein conformation.

## Results

### Clinical features

**Figure 1:**
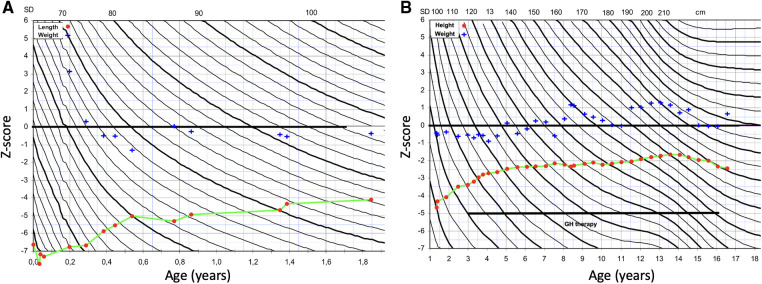
Growth charts of the patient. (**A**) Z-score for length (red dot) and weight-for-length (blue cross) at 0–2 years of age. (**B**) Z-score for height (red dot) and weight-for-height at 1–16 years of age. Growth hormone (GH) therapy was started at 3 years of age.

We report a 16-year-old boy born at 37 + 2 weeks of gestation to healthy parents of Finnish descent. Fetal growth failure was diagnosed by ultrasound at gestational week 19 and he was born very small for gestational age with birth length 38.5 cm (−6.1 SDS), birth weight 1480 g (−4.3 SDS) and a head circumference of 30.5 cm (−3.2 SDS). He had relative macrocephaly, with head circumference 2.9 SDS above birth length SDS. Weight of the placenta was 260 g, which is remarkably lower than the 3rd centile placental weight reported in a Scandinavian cohort (426 g for 261 gestational days, male sex and multiparity) (19). Figure 1 presents the growth charts of the patient. From the age of 3 months, he experienced spontaneous catch-up growth; the length SDS was −6.6 at 3 months, −5.5 at 6 months, and −3.4 at 3 years of age. Postnatally he suffered from severe feeding difficulties. Weight development with both nutritional and caloric supplement was good. He received gastric tube feeding from the age of 5 months and had a percutaneous endoscopic gastrostomy inserted at 8 months. He presented with a triangular-shaped face, small chin, thin lips, and 5th finger clinodactyly. He had no body asymmetry. Brain ultrasound and echocardiogram were normal. He had normal chromosomes, and genetic testing for GRACILE syndrome and Mulibrey nanism were normal. Silver-Russell Syndrome was suspected early neonatally. He was tested for UPD(7)mat and 11p15 methylation with normal results. A clinical diagnosis of SRS was made. He did not present with any cognitive, motor or speech delay. From the age of 3 years, he received recombinant human growth hormone (rhGH) treatment to increase adult height. Pretreatment investigations presented normal growth hormone (GH), high to normal serum insulin-like growth factor 1 (IGF1) and normal insulin-like growth factor-binding protein 3 (IGFBP3) levels. During GH treatment, serum IGF1 level was consistently at a high range, with normal to low GH doses. GH therapy accelerated the growth velocity; height SDS was −3.4 at the start of therapy, −2.8 after 1 year of treatment, −2.5 after 2 years of treatment, and −1.7 at pubertal onset. Pubertal timing was normal. Testicular size was small. In early childhood serum FSH, LH, testosterone and inhibinB were measured twice and the levels were normal. Testicular growth and virilization proceeded close to normal. At the end of the puberty, testicular size was in low normal range (testicular length 30 mm) and the secondary sex characteristics had reached stage P5G4. The attained final height at age 16 years was 160 cm (−2.2 SDS).

### Genetic findings

The custom-made targeted exome sequencing included sequence and copy number variation analyses of 566 genes. Median read depth for the analysis was 234-fold, and 99.78% of target nucleotides were covered with >20-fold read depth. CNV analysis did not detect any known disease-causing or novel CNVs that were considered pathogenic. A novel *de novo* heterozygous missense variant NM_000612.5 (*IGF2*) c.122T > G, p.Leu41Arg was identified in the patient. The identified variant has not been observed in gnomAD, 1,000 Genomes, or Sequencing Initiative Suomi database (SISu), and has not been reported in dbSNP, ClinVar, or HGMD. This variant was predicted as pathogenic/damaging by SIFT, MutationTaster2, M-CAP, MVP, Provean, REVEL and PrimateAI. Polyphen2 predicted the variant as probably damaging. This variant has a CADD score 27.4.

**Figure 2:**
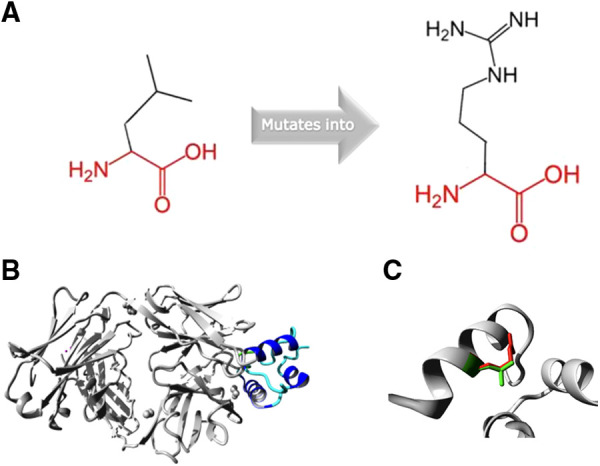
(**A**) Schematic structure of the original and the mutant amino acid. (**B**) Overview of the protein in ribbon-presentation. The protein is coloured by element; α-helix = blue and random coil = cyan. (**C**) Close-up of the mutation. The protein is coloured grey, the side chains of the wild-type (green) and the mutant residue (9).

The missense variant is located in the disulfide bond domain of IGF2 and affects a highly conserved residue (PhyloP 100-way score of 7.912). Analysis of the mutant protein by HOPE showed that the mutant residue is bigger, more hydrophilic and introduces a positive charge compared to the smaller, neutral charged wildtype residue (Figure 2).

**Figure 3:**
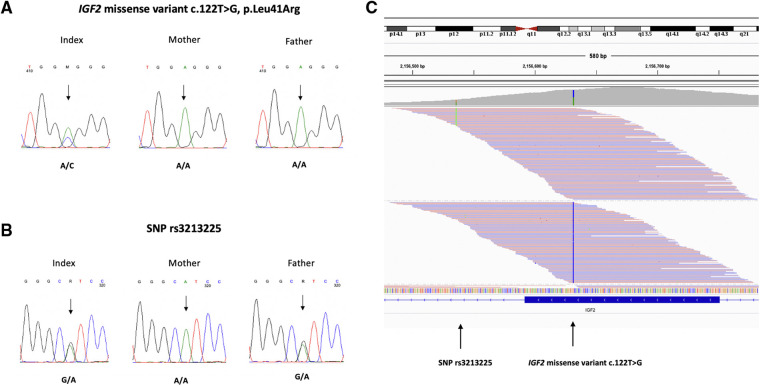
(**A**) Chromatograms of direct sequence analysis of *IGF2* gene showing the missense variant c.122T > G, p.Leu41Arg in the patient (**B**) the SNP rs3213225 in the patient and father (**C**) IGV view of the patient's sequence data shows that the reads with SNP alternative allele A (in green) and the missense variant c.122T > G (in blue) are on different reads. The father is heterozygous (G/A) for the SNP and the mother is homozygous (A/A). The reads with the SNP reference allele G, that also carry the missense variant c.122T > G, must be paternal.

The *IGF2* missense variant was confirmed by Sanger sequencing and was absent in the parents (Figure 3A). We investigated the parental origin of the *IGF2* missense variant by examining a common SNP (rs3213225) located 96 bp away from the variant. The child and father were heterozygous (G/A) for the SNP and the mother was homozygous (A/A) (Figure 3B). By exploring the reads in the Integrative Genomics Viewer (IGV), we found that the reads with SNP reference allele G also carried the missense variant indicating that the missense variant occurred on the paternal allele (Figure 3C).

Table 1 presents the clinical features of our patient and the patients with previously reported *IGF2* variants.

**Table 1:** Clinical characteristics in our patient and patients with previously reported *IGF2* variants (6, 8, 11–18).

	Our patient	Previously reported patients
Prenatal growth failure/SGA^a^	Yes	19/19 (100%)
Postnatal growth failure^a^	Yes	20/20 (100%)
Feeding difficulties/low BMI^a^	Yes	18/18 (100%)
Triangular face	Yes	17/18 (94%)
Prominent forehead^a^	Yes	16/18 (89%)
Relative macrocephaly at birth^a^	Yes	16/18 (89%)
Developmental delay/intellectual disability	No	12/15 (80%)
Clinodactyly	Yes	15/19 (79%)
Low set ears	No	8/13 (62%)
Cleft palate	No	7/14 (50%)
Cardiovascular anomalies	No	9/18 (50%)
Body asymmetry^a^	No	4/18 (22%)
Syndactyly	No	4/18 (22%)

^a^ Included in the Netchine-Harbison Clinical Scoring System.

## Discussion

SRS can be caused by several different genetic mechanisms including epimutations, uniparental disomy, rare pathogenic single nucleotide variants and copy number variants. About 40% of patients with SRS phenotype remain without a molecular diagnosis. Recently, next generation sequencing has been suggested as a useful tool to improve the identification of novel variants associated with SRS (6, 8). We describe a novel *de novo IGF2* missense variant identified by targeted exome sequencing in a patient with a clinical diagnosis of SRS. The missense variant was predicted pathogenic by multiple *in silico* tools and affects a highly conserved residue. The variant was also confirmed to be on the paternal allele, consistent with the paternal expression pattern of *IGF2*.

To our knowledge, only 16 pathogenic/likely pathogenic *IGF2* variants have been reported in 20 patients with SRS-like phenotype but the prevalence of *IGF2* variants in large SRS populations remains unknown (6, 8, 11–18). The previously described *IGF2* variants include eight missense, three splicing, three frameshift and two stop mutations. All patients presented with pre- and postnatal growth restriction and feeding difficulties. Our patient had severe prenatal growth restriction and feeding difficulties, but he had spontaneous catch-up growth from the age of 3 months. He had stable weight development though he required feeding measures. Postnatal catch-up growth is not usually seen in patients with SRS, including patients with *IGF2* variants. 90% of the patients with *IGF2* variants had relative macrocephaly, a prominent forehead, and a triangular face. These features were also observed in our patient. He did not present with any developmental delay or intellectual disability, which has been reported in 80% of the patients with *IGF2* variants. However, in five out of twenty patients, there was no information about developmental delay/intellectual disability. Notably, our patient had high serum IGF1 level, which has also been reported in other patients with *IGF2* variants (11, 14, 18). Masunga et al. compared clinical features and endocrine findings between patients with *IGF2* variants and those with *H19*/*IGF2*:IG-DMR epimutations and found that *IGF2* variants were associated with higher serum IGF1 level. They also reported that *IGF2* variants were associated with high frequency of cardiovascular anomalies and developmental delay, and low frequency of body asymmetry compared to *H19*/*IGF2*:IG-DMR epimutations (14). Our patient had no body asymmetry, which may be one of the clinical features that best differentiates *IGF2* variants from SRS caused by epigenetic changes.

Genetic counseling is recommended for patients with SRS and their families. The recurrence risk depends on the underlying cause of SRS and the sex of the transmitting parent. In most cases, only one child is affected and the recurrence risk for SRS in cases of *de novo* loss of paternal methylation of H19/IGF2 ICR1 or UPD(7)mat is very low. However, when SRS is caused by CNVs or pathogenic variants the recurrence risk can be up to 50%. In our case with a *de novo* pathogenic variant in *IGF2*, the recurrence risk is 50% for the offspring of the proband. Genetic testing is important for accurate genetic counseling.

IGF2 is an important prenatal growth factor and regulates the fetal demand and placental supply of nutrients (20). Small hypoplastic placentas and decreased levels of IGF2 have been found in patients with SRS (12, 14, 18). A hypoplastic placenta was also found in our patient. A recent mouse study revealed a direct role for the imprinted *igf2*-*igf2r* axis on matching placental development to fetal growth and showed that IGF2 produced by the fetus plays an important role in controlling placental microvasculature and trophoblast morphogenesis in late gestation (21). They suggest that poor placentation in fetal growth restriction may be due to deficient microvasculature expansion caused by decreased IGF2 signaling from the fetus. Another animal study proposed that growth anomalies in IGF2-dependent SRS can be detected prenatally by measuring IGF2 peptide levels in the amniotic fluid and prevented by prenatal genetic rescue targeting IGF2 (22). More studies are needed to investigate IGF2-based prenatal diagnosis and intervention strategies for IGF2-dependent SRS.

In conclusion, our finding expands the molecular and phenotypic spectrum of disease-causing variants in SRS and highlights the importance of screening for pathogenic variants in *IGF2*, especially when common diagnostic tests for SRS are negative. Next generation sequencing will likely increase the diagnostic yield of SRS. Whole exome and genome sequencing can identify novel genes associated with SRS or genes associated with differential diagnosis of SRS. Molecular diagnosis is required for specific genetic counseling and targeted clinical management.

## Data Availability

The datasets for this article are not publicly available due to concerns regarding participant/patient anonymity. Requests to access the datasets should be directed to the corresponding author.
